# Simulation of *Escherichia coli* Dynamics in Biofilms and Submerged Colonies with an Individual-Based Model Including Metabolic Network Information

**DOI:** 10.3389/fmicb.2017.02509

**Published:** 2017-12-13

**Authors:** Ignace L. M. M. Tack, Philippe Nimmegeers, Simen Akkermans, Ihab Hashem, Jan F. M. Van Impe

**Affiliations:** BioTeC+, Department of Chemical Engineering, KU Leuven, Ghent, Belgium

**Keywords:** multiscale modeling, individual-based modeling, metabolomics, *E. coli*, biofilm dynamics

## Abstract

Clustered microbial communities are omnipresent in the food industry, e.g., as colonies of microbial pathogens in/on food media or as biofilms on food processing surfaces. These clustered communities are often characterized by metabolic differentiation among their constituting cells as a result of heterogeneous environmental conditions in the cellular surroundings. This paper focuses on the role of metabolic differentiation due to oxygen gradients in the development of *Escherichia coli* cell communities, whereby low local oxygen concentrations lead to cellular secretion of weak acid products. For this reason, a metabolic model has been developed for the facultative anaerobe *E. coli* covering the range of aerobic, microaerobic, and anaerobic environmental conditions. This metabolic model is expressed as a multiparametric programming problem, in which the influence of low extracellular pH values and the presence of undissociated acid cell products in the environment has been taken into account. Furthermore, the developed metabolic model is incorporated in MICRODIMS, an in-house developed individual-based modeling framework to simulate microbial colony and biofilm dynamics. Two case studies have been elaborated using the MICRODIMS simulator: (i) biofilm growth on a substratum surface and (ii) submerged colony growth in a semi-solid mixed food product. In the first case study, the acidification of the biofilm environment and the emergence of typical biofilm morphologies have been observed, such as the mushroom-shaped structure of mature biofilms and the formation of cellular chains at the exterior surface of the biofilm. The simulations show that these morphological phenomena are respectively dependent on the initial affinity of pioneer cells for the substratum surface and the cell detachment process at the outer surface of the biofilm. In the second case study, a no-growth zone emerges in the colony center due to a local decline of the environmental pH. As a result, cellular growth in the submerged colony is limited to the colony periphery, implying a linear increase of the colony radius over time. MICRODIMS has been successfully used to reproduce complex dynamics of clustered microbial communities.

## Introduction

In their natural or industrial settings, many bacterial species form clustered communities, such as biofilms or colonies, rather than living in a free-swimming planktonic state. Living as a community often confers specific advantages, such as antibiotic resistance and immune evasion due to horizontal gene transfer, resistance against flow shear forces and persistence in dynamic and stressing environments due to metabolic differentiation (Costerton, 1995; Costerton et al., 1999). This metabolic differentiation is the result of diffusion limitations and concomitant gradients in the nutrient, oxygen and metabolic waste product concentrations. These concentration gradients are induced by nutrient/oxygen consumption and the secretion of metabolic waste products such as acetic, formic, and lactic acid by the relatively densely packed cells (Stewart and Franklin, 2008).

Microbial colony and biofilm development is a significant issue in food industry. Biofilm formation on food equipment surfaces constitutes a major contamination source of the food products. Surface colony growth occurs when food surfaces are exposed to these microbial contamination sources of spoiling or pathogenic organisms. In mixed or coagulated food products, such as minced meat or cheese, these microbial contaminants can penetrate the food interior during the production process, resulting in submerged colony growth (Wimpenny et al., 1995). Cellular growth in clustered communities may also be used intentionally to obtain specific beneficial effects, such as biodegradation or synthesis by the use of catalytic biofilms (Benedetti et al., 2016).

This article focuses on the simulation of *Escherichia coli* biofilm and colony growth dynamics. *E. coli* is a particularly dangerous food pathogen for young, elderly and immunocompromised people causing gastrointestinal disorders, renal failure or even death (Rowe, 2009). An increasing trend of *E. coli* infections has been observed in the EU from 2009 to 2013, possibly due to an increased awareness after the large outbreak of EHEC O104:H4 in 2011 (EFSA and ECDC, 2015). In addition, as a facultative anaerobe, *E. coli* is able to survive both in the presence and absence of oxygen, increasing the risk of food contamination. Therefore, accurate quantitative microbial risk assessment is indispensable to guarantee microbial food safety in the whole food production and distribution chain. For this purpose, mathematical models are developed in predictive microbiology to describe and predict microbial dynamics in food products as a function of environmental conditions resembling food processing and storage (Buchanan, 1993).

Traditionally, predictive models are (semi-)empirically based on data of microbial dynamics in well-mixed liquids and only consider the average microbial population dynamics at a macroscopic scale. However, as a result of the heterogeneous environmental conditions in mature biofilms and colonies, and due to the concomitant metabolic differentiation among the constituting cells, the individual microbial cell is the most intuitive modeling level. In individual-based models (IbM), the individuals/agents of a population are described as discrete, unique, and autonomous entities (Grimm and Railsback, 2005; Railsback and Grimm, 2012). This enables the modeler to include individual variability, directed or local interactions of agents with the surrounding medium or other agents, and adaptive physiological behavior. Population dynamics are not modeled explicitly, but emerge from the behavior of the individuals and their interactions with the environment and each other. As suggested above, in predictive microbiological IbMs, the microbial cell is taken as the individual modeling unit (Ginovart et al., 2002; Standaert et al., 2004; Dens et al., 2005; Prats et al., 2006; Verhulst et al., 2011; Ferrier et al., 2013; Tack et al., 2014, 2015).

Despite the specific advantages of IbMs, this kind of models is notorious for its complex structure (Grimm, 1999; Grimm et al., 1999). While traditional predictive models only consist of a limited set of equations, IbMs contain a multitude of mathematical and logical rules grouped in submodels, each representing a major and more or less independent process of the real-life system. This complexity can make IbMs computationally intensive and slow to run, hard to comprehend and communicate, data hungry, prone to overfitting, difficult to calibrate, and laborious to test. In the microbial systems considered in this work, the most complex process is the metabolism of the *E. coli* cells, which is determined by a myriad of possible intracellular reaction pathways. Therefore, it is necessary to develop a non-complex, yet accurate metabolic model, valid under the environmental conditions in our case studies.

Information on the individual cell metabolism could be included in IbMs by metabolic flux balance analysis (FBA) with genome-scale models (Palsson, 2006). To represent the specific microbial growth rate or the secretion rates of major cell products as a function of nutrient and oxygen consumption, phenotypic phase planes (PhPPs) can be constructed by performing FBAs at varying specific cellular nutrient and oxygen uptake rates. However, this would result in long run times due to the thousands/millions of cells in IbMs and the myriad of intracellular pathways in FBA. In addition, FBAs determine metabolic flux distributions by optimizing a certain cellular objective (e.g., maximization of biomass or metabolite production) which is often unknown, especially when the cell is exposed to stressing environmental conditions (Feist and Palsson, 2010). Therefore, the PhPPs are approximated in this article with a low-complexity linear model that contains the intracellular information from the FBAs without explicitly incorporating it. However, the PhPPs to which the linear model is calibrated are only valid when cells aim to maximize their growth. To account for deviations from growth-optimal conditions in the culture environment, growth inhibition and metabolic shifts due to low pH values and the presence of weak acid cell products in the environment are incorporated in the linear model.

Finally, the linear metabolic model is incorporated in MICRODIMS, an in-house developed IbM (Verhulst et al., 2011; Tack et al., 2015). In this way, MICRODIMS is applicable as a virtual laboratory to explore the behavior of *E. coli* cells on/in food products under various combinations of heterogeneous environmental conditions. Two case studies are elaborated in detail: (i) two-dimensional biofilm growth on abiotic food equipment surfaces, and (ii) three-dimensional submerged colony growth occurring in mixed or coagulated semi-solid food products. In both case studies, oxygen limitations emerge in mature microbial structures due to diffusion limitations, leading to local pH drops as a result of the cellular secretion and accumulation of weak acid substances. The resulting metabolic differentiation among the cells influences the global population dynamics: low pH values and cell lysis at the substratum surface lead to biofilm detachment, while submerged colonies are characterized by the emergence of a central no-growth zone influencing the colony radius growth.

## Materials and methods

In the first part of this section, the basic concepts of developing a non-complex metabolic model for *E. coli* are explained. These concepts have already been partly described in Tack et al. (2014). Secondly, the structure of the IbM in which the metabolic model is incorporated is described according to the ODD (Overview, Design concepts, and Details) protocol of Grimm et al. (2006, 2010).

### Development of a non-complex metabolic model for *E. coli*

The non-complex metabolic model that is incorporated in the IbM is based on systems biology concepts. These concepts and the derivation of the developed metabolic model will be explained in the following subsections. Furthermore, the dependency of the metabolic model outputs (i.e., the specific cellular growth rate and the secretion rates of the main cell products) on the environmental pH and concentrations of weak acid cell products is explained.

#### Flux balance analysis

In mathematical terms, the intracellular metabolic pathways and the exchange reactions with the extracellular environment are contained within the exchange stoichiometric matrix **S**_exch_ (Palsson, 2006):

(1) $$\begin{array}{r} \frac{\text{d}\text{x}}{\text{d}t}={\text{S}}_{\text{exch}}\left(\begin{array}{c} \text{v}\\ \text{q} \end{array}\right), \end{array}$$

with **x** the concentrations of the involved metabolites, and **v** and **q** the metabolic fluxes through respectively the intracellular and exchange reactions.

Flux balance analysis (FBA) determines a steady-state solution of Equation (1) by optimizing a specific cellular objective function *J*, leading to the following optimization problem (Palsson, 2006):

(2) $$\begin{array}{r} \underset{\text{v},\text{q}}{\min}\left[J=\text{w}\cdot \left(\begin{array}{c} \text{v}\\ \text{q} \end{array}\right)\right], \end{array}$$

subject to

(3) $$\begin{array}{r} {\text{S}}_{\text{exch}}\left(\begin{array}{c} \text{v}\\ \text{q} \end{array}\right)=0, \end{array}$$

(4) $$\begin{array}{r} v_{i,min}\leq v_{i}\leq v_{i,max}, \end{array}$$

(5) $$\begin{array}{r} q_{i,min}\leq q_{i}\leq q_{i,max}. \end{array}$$

In Equation (2), the weights **w** are defined by the intended cellular objective. The constraints on the kinetic rates of the intracellular reactions (*v*_*i, min*_ and *v*_*i, max*_) and the physicochemical constraints on the external fluxes due to environmental conditions (*q*_*i, min*_ and *q*_*i, max*_) are taken into account in Equations (4, 5) respectively.

#### Phenotypic phase plane analysis

**Figure 1:**
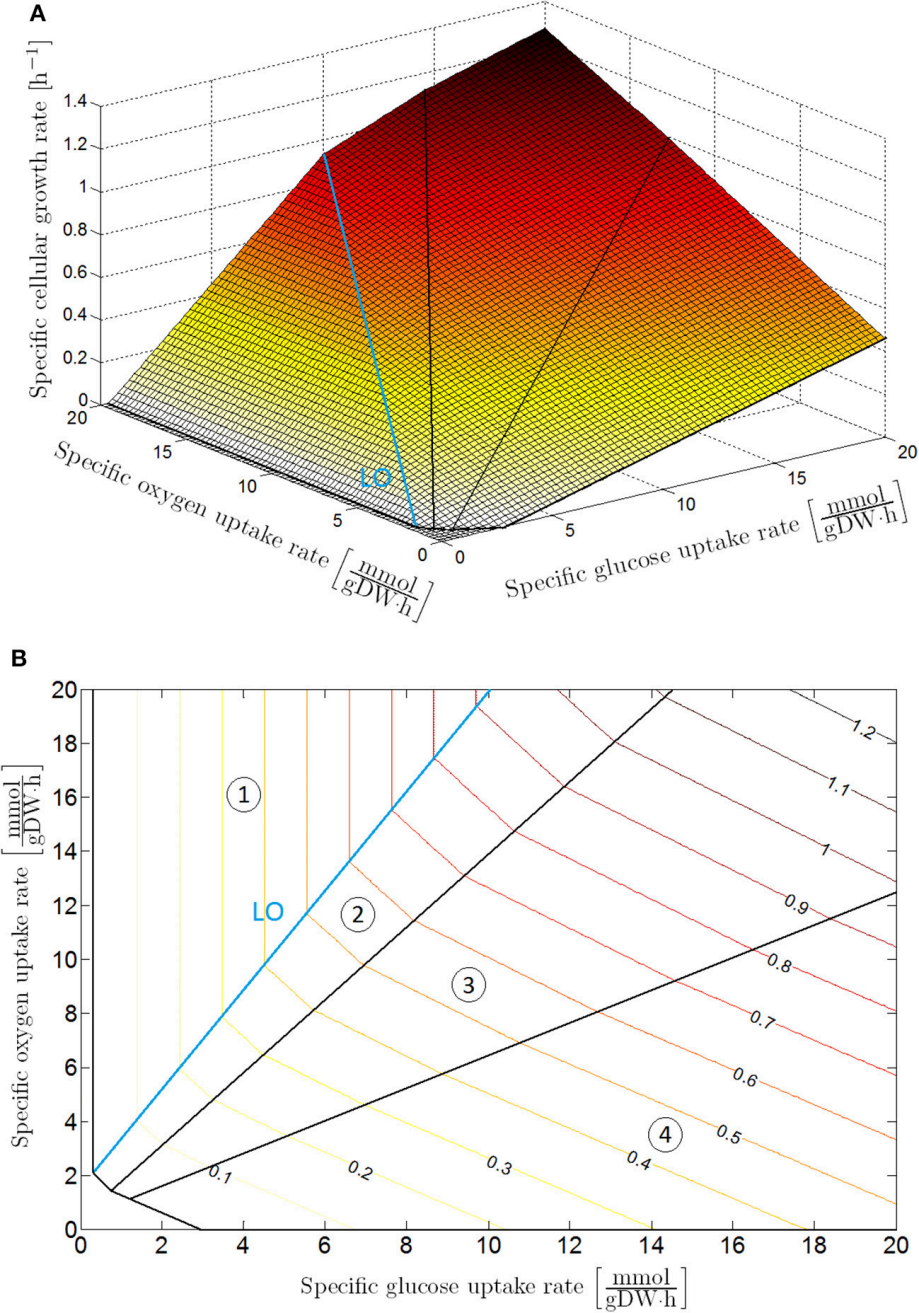
Phenotypic phase plane analysis: specific cellular growth rate as a function of specific glucose and oxygen uptake rates with maximization of biomass growth as cellular objective, presented as **(A)** 3D plot and **(B)** contour plot. The phenotypic phase plane consists of four phases, each representing a different metabolic regime. In Sector 1 glucose is completely converted to CO_2_ through the tricarboxylic (TCA) cycle. The other sectors are characterized by the secretion of weak acid cell products in the cellular environment: acetic acid in Sector 2; acetic and formic acid in Sector 3; acetic acid, formic acid and ethanol in Sector 4. On the boundary between Sector 1 and 2, glucose is converted to biomass at a maximal observed yield. For this reason, this boundary is indicated as the line of optimality (LO).

Flux balance analysis is an accurate tool to determine the specific cellular growth rate and secretion rates of the main weak acid cell products for a known specific glucose and oxygen uptake rate. To examine the metabolic regimes of *E. coli* under different glucose and oxygen availability conditions, a phenotype phase plane (PhPP) analysis can be carried out by executing multiple FBAs for a range of specific glucose and oxygen uptake rates (Edwards et al., 2001), as illustrated in Figure 1. For these FBAs, the genome-scale model iAF1260 of Feist et al. (2007) has been used, and it has been assumed that the cell aims to maximize its biomass. The metabolic operation of the cell is retrieved from the PhPP by maximizing the specific cellular growth rate as a function of the specific glucose and oxygen uptake rate:

(6) $$\begin{array}{r} \underset{q_{G},q_{O}}{\max}\left[J=\mu \left(q_{G},q_{O}\right)\right] \end{array}$$

subject to

(7) $$\begin{array}{r} 0\leq q_{G}\leq q_{G,max}\cdot \frac{C_{G}}{K_{G}+C_{G}}, \end{array}$$

(8) $$\begin{array}{r} 0\leq q_{O}\leq q_{O,max}\cdot \frac{C_{O}}{K_{O}+C_{O}}. \end{array}$$

The upper constraints for the glucose and oxygen uptake rate are determined according to the Monod kinetic law (Monod, 1942). In these constraints, *q*_*G, max*_ $\left[\frac{\text{mol}}{\text{gDW}\cdot \text{h}}\right]$ and *q*_*O, max*_ $\left[\frac{\text{mol}}{\text{gDW}\cdot \text{h}}\right]$ are kinetically the maximal specific uptake rates for glucose and oxygen (where DW is the abbreviation of dry weight, mol the indication of mole, and h the abbreviation of hour), *C*_*G*_ [mol/L] and *C*_*O*_ [mol/L] the extracellular glucose and oxygen concentrations (with L the abbreviation of liter), while *K*_*G*_ [mol/L] and *K*_*O*_ [mol/L] are the Monod half-saturation constants for respectively glucose and oxygen. The optimization problem in Equation (6) is straightforward to solve as the PhPP in Figure 1 is a monotonically increasing function of both the glucose and oxygen uptake rate, implying that the specific glucose and oxygen uptake rates are equal to the upper boundaries in Equations (7, 8). Values for the above-mentioned parameters can be found in Table 1.

**Table 1:** Parameter values.

**Parameter**	**Value**	**Reference**
*q*_*G*, max_	9.02·10^−3^ mol/(gDW·h)	Portnoy et al., 2010
*q*_*O*, max_	16.49·10^−3^ mol/(gDW·h)	Portnoy et al., 2010
*K*_*G*_	2.994·10^−6^ mol/L	Ihssen et al., 2007
*K*_*O*_	0.121·10^−6^ mol/L	Stolper et al., 2010

#### A linear metabolic model for *E. coli* under reference environmental conditions

As the PhPP contains much information about intracellular metabolic fluxes from its constituting FBAs, it is not appropriate to incorporate it in its original form into an IbM. Multiple evaluations of the whole intracellular reaction network would result in an excessive increase of the required IbM simulation run time. For this reason, the planes in the PhPP at a specific oxygen uptake rate are described by means of the linear growth law of Pirt (Schulze and Lipe, 1964; Pirt, 1965):

(9) $$\begin{array}{r} \mu =\left(q_{G}-m_{G}\right)\cdot Y_{X/G}, \end{array}$$

with *q*_*G*_ $\left[\frac{\text{mol}}{\text{gDW}\cdot \text{h}}\right]$ the specific glucose uptake rate, *m*_*G*_ $\left[\frac{\text{mol}}{\text{gDW}\cdot \text{h}}\right]$ the maintenance coefficient, and *Y*_*X*/*G*_ [gDW/mol] the biomass yield coefficient on glucose. The secretion rates of the main acid metabolites (acetic, formic, and lactic acid) are expressed similarly:

(10) $$\begin{array}{r} q_{i}=Y_{i/G}\cdot q_{G}+q_{i}^{0}, \end{array}$$

where *q*_*i*_ $\left[\frac{\text{mol}}{\text{gDW}\cdot \text{h}}\right]$ is the specific secretion rate of metabolite *i*, $q_{i}^{0}$ is the specific secretion rate at a zero glucose uptake rate and *Y*_*i*/*G*_ [mol/mol] the production yield of metabolite *i* on glucose.

#### The influence of pH and weak acids on the *E. coli* metabolism

In the optimization problem in Equations (6–8), it is assumed that the cell aims to maximize its growth. This assumption is only valid for a non-stressing reference environment, i.e., a neutral M9 minimal medium enriched with glucose at 37°C. However, the cellular secretion of weak acid metabolites under oxygen-limited conditions in microbial biofilms and colonies constitutes an inhibiting factor for cellular growth and survival. The inhibiting effect of weak acid cell products on cellular growth is 2 fold: (i) dissociation of acid metabolites in the food environment leads to a decrease of the extracellular pH, and (ii) reintrusion of the lipophilic undissociated cell products into the cell results in an intracellular pH drop.

Under the stressing conditions of low extracellular pH values and the presence of undissociated acid cell products, the cellular objective changes to maximize survival chances. As a matter of fact, the cellular objective needs to be modified as

(11) $$\begin{array}{r} \underset{q_{G},q_{O}}{\max}J\left(q_{G},q_{O},pH,\left[U_{i}\right]\right), \end{array}$$

restating the optimization problem in Equations (6–8) as a multiparametric programming problem. However, the exact mathematical formulation of the cellular objective is unfortunately not known. For this reason, a more pragmatic approach is required.

Synergistic effects of low extracellular acidity and undissociated acid cell products in the environment can be taken into account in the maintenance coefficient in Equation (9):

(12) $$\begin{array}{r} m_{G}=m_{G,ref}+A\cdot \frac{\left[{\text{H}}^{+}\right]-10^{-7}}{{\left[{\text{H}}^{+}\right]}_{min}-10^{-7}}+B\cdot \underset{i}{\sum }\frac{\left[{\text{U}}_{i}\right]}{{\left[{\text{U}}_{i}\right]}_{min}}. \end{array}$$

In this expression, the maintenance coefficient consists of three terms: (i) the reference maintenance coefficient *m*_*G, ref*_ that can be derived from the reference PhPP in Figure 1, (ii) additional maintenance requirements due to an increase of the extracellular proton concentration [H^+^], and (iii) a supplementary effect of weak acid cell products in their undissociated form [U_*i*_]. Microbial growth stops when the proton concentration reaches a critical value [H^+^]_*min*_. Analogous minimum inhibitory concentrations [U_*i*_]_*min*_ exist for the undissociated acid cell products. The mathematical constants *A* and *B* are calculated by replacing this expression for the maintenance coefficient in Equation (9).

The increase of the maintenance coefficient due to low extracellular pH values and the presence of undissociated acid cell products does not only affect the microbial biomass growth, but also the secretion of metabolic products in Equation (10):

(13) $$\begin{array}{r} q_{i}=Y_{i/G}\cdot q_{G}+\frac{m_{G}}{m_{G,ref}}\cdot q_{i}^{0}, \end{array}$$

### ODD description of the developed IbM

The developed metabolic model is incorporated in the MICRODIMS IbM (Verhulst et al., 2011; Tack et al., 2015). This in-house developed IbM is adapted and extended to simulate the dynamics of microbial biofilms and submerged colonies. In this section, a general overview of the MICRODIMS simulator is described according the to first part of the standard ODD protocol of Grimm et al. (2006, 2010). Details about the specific MICRODIMS versions for the two considered case studies are included in the next section.

#### Model purpose

The purpose of the new MICRODIMS versions is the simulation of chemical gradients and the resulting metabolic differentiation in *E. coli* biofilms and submerged colonies. The influence of this metabolic differentiation on the development of mature biofilms and colonies is investigated as well.

#### Entities, state variables, and scales

Microbial systems consist of two kinds of agents: the microbial cells and their surrounding environment.

The microbial cells contain the same state variables as in the previous MICRODIMS versions (Tack et al., 2015): cell mass and radius, spatial coordinates, maximum specific glucose and oxygen uptake rates and a list variable with information about the ongoing DNA replication cycles. In addition, an inclination vector is introduced to take the rod shape and orientation of *E. coli* into account.

The liquid environment is modeled as a two-dimensional space for the biofilm simulations, while the submerged colonies are simulated in a three-dimensional food environment. In both cases, the environment is discretized as a spatial grid. Each of the grid units is associated with a glucose, oxygen, acetic acid and formic acid concentration. To simulate chemical gradients at a microscopic level, the size of a grid unit needs to be in the same order of magnitude as a microbial cell, viz., 6 μm. The whole space has a dimension of 300 μm.

#### Process overview and scheduling

**Figure 2:**
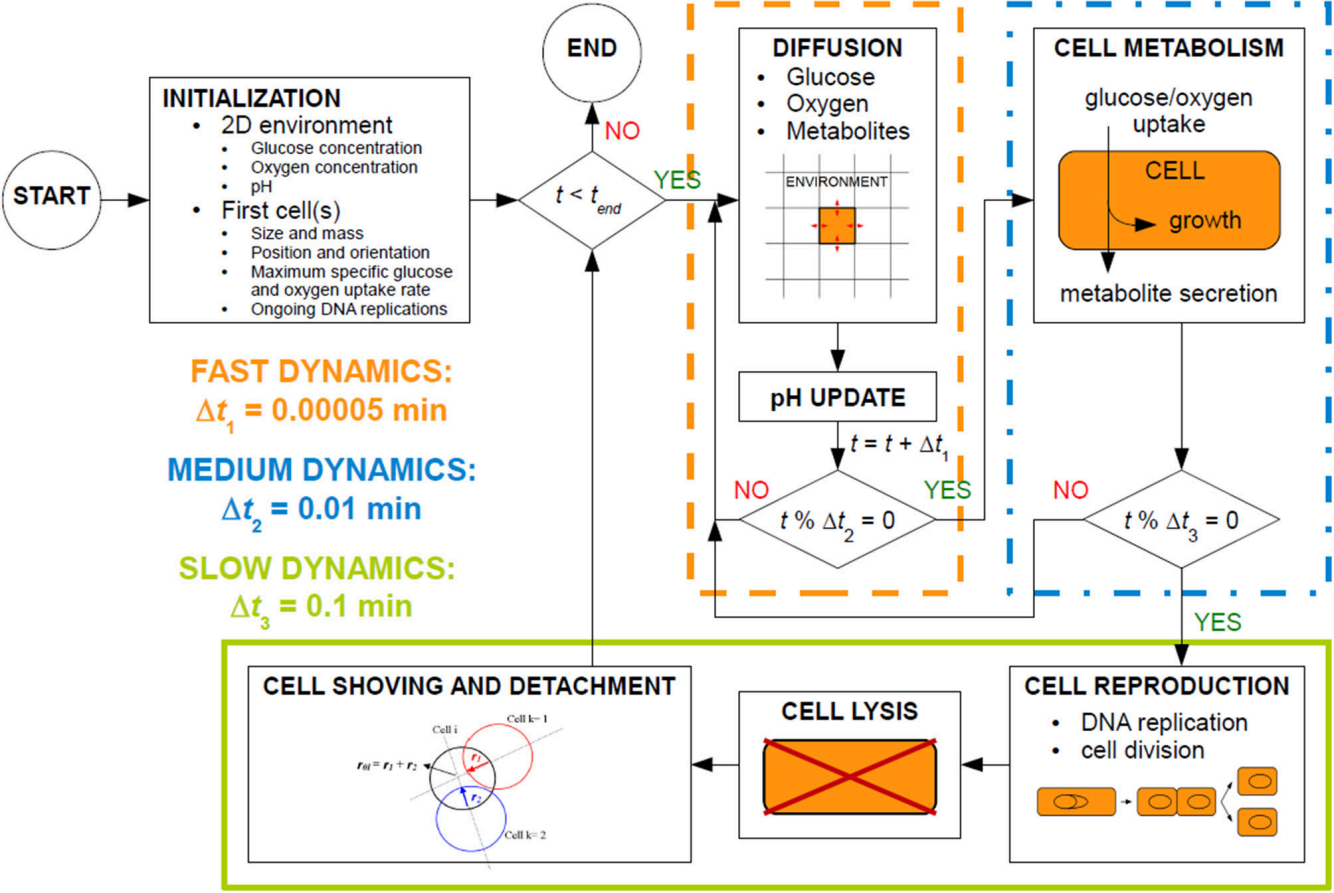
Process schedule of the MICRODIMS model.

MICRODIMS consists of several interlinked subprocesses, as illustrated in Figure 2. These subprocesses exhibit different time dynamics (Picioreanu et al., 1999) and are executed at different time steps. In MICRODIMS, three time steps are used: (i) Δ*t*_1_ = 0.00005 min, a very short time step for the fast diffusion processes and the update of local pH values, (ii) Δ*t*_2_ = 0.01 min, an intermediate time interval for the metabolic processes of nutrient and oxygen uptake, biomass growth and metabolite secretion, and (iii) Δ*t*_3_ = 0.1 min, the long time step for slow processes such as cell reproduction, cell lysis, and cell movement. This cell movement consists of a shoving mechanism to avoid spatial overlap between the cells, and a detachment process for loosely bound biofilm cells. After the initiation of the simulation, all the subprocesses are executed consecutively as presented in the simulation scheme in Figure 2.

#### Software

The MICRODIMS IbM has been implemented in the MASON multiagent simulation toolkit in Java (Luke et al., 2003, 2004, 2005). The strict separation between the execution of the model and graphical output increases the simulation speed in comparison to other IbM simulation toolkits, such as Repast Simphony.

## Results

### Case study I: biofilm growth

Over the last years, several models have been developed for the description of single species biofilm structures using information of genome-scale metabolic models (Biggs and Papin, 2013; Chen et al., 2016; Bauer et al., 2017). However, these models basically rely on one determining factor to explain the simulated biofilm morphologies at a mesoscopic level, namely the diffusion of nutrients/oxygen into and the diffusion of metabolic waste products out of the biofilm. In addition, the modeling unit in these cellular automaton models is the local microbial concentration in a small square of the environment. This spatial resolution that is used to model the microbial cells, is too coarse to simulate morphological phenomena at a finer microscopic scale, such as the formation of cellular chains at the outer biofilm surface. To take these microscopic events into account, each cell needs to be modeled as a separate discrete entity in a realistic individual-based model, where the modeling unit is the microbial cell itself and direct microscopic interactions between the microbial cells (such as intercellular adhesion) can be taken into account. The emergence of these microscopic morphological phenomena in *E. coli* biofilms is described in this case study.

#### Model details

Besides the incorporation of the developed metabolic model, other submodels in the basic MICRODIMS module of Verhulst et al. (2011) needed to be slightly adapted or included to simulate the characteristic dynamics of biofilms on food processing surfaces. In this subsection, these adaptations, and extensions are described in more detail.

##### Initialization and boundary conditions

The initial environment is neutral and does not contain any initial weak acid cell products. Initially, the oxygen concentration is taken to be the saturated oxygen concentration in water (6.73 mg/L) in order to clearly demonstrate the transition from aerobic to anaerobic environmental conditions in the biofilm. Furthermore, the environment has a glucose concentration of 0.1 g/L in order to ensure that the simulation starts with a metabolic regime in region 1 of the PhPP (see Figure 1). In reality, the initial glucose concentration is typically higher (1 g/L) implying that the simulation would start in metabolic regime 2, 3, or 4 with a very high production of weak acid cell products already in the initial stages of the simulation. Consequently, the simulation would not demonstrate the full transition from aerobic to anaerobic conditions. In the environment, biofilm growth starts from three cells randomly situated at the substratum surface. This substratum is situated at the lower environmental boundary. It is modeled by means of a Neumann boundary condition, i.e., any chemical gradients or fluxes are absent at this insulating surface. In contrast, the opposite upper boundary of the environment is in contact with the bulk medium and is characterized by a Dirichlet boundary condition with constant concentrations. In this way, the bulk medium is represented as an infinite reservoir of nutrients and oxygen, and an infinite sink for metabolic waste products. The remaining two side boundaries are wrapped toward each other, creating periodic boundary conditions.

##### Diffusion

Diffusion is modeled according to the second law of Fick:

(14) $$\begin{array}{r} \frac{\partial C_{i}}{\partial t}=D_{i}\cdot \left(\frac{\partial^{2}C_{i}}{\partial x^{2}}+\frac{\partial^{2}C_{i}}{\partial y^{2}}\right), \end{array}$$

with *C*_*i*_ [mol/L] the concentration of substance *i* in the liquid phase, $D_{i}$ [μm^2^/min] the diffusion coefficient of substance *i*, *x* [μm] and *y* [μm] the spatial dimensions, and *t* [min] the temporal dimension. This equation has no analytical solution in combination with the initial and boundary conditions defined in the previous paragraph. For this reason, it is discretized according to an explicit Forward-Time Central-Space (FTCS) numerical scheme (Roache, 1972). To incorporate the restrictive effect of EPS and surrounding microbial cells on the diffusion processes, the diffusivity $D_{i}$ is deliberately decreased by a factor of 50 in the biofilm to provide a good match between the simulated biofilm morphologies and experimentally observed biofilm structures, as there are unfortunately no direct experimental data available for this decrease in diffusivity.

##### pH update

Local pH values are calculated from the acid cell product concentrations and their dissociation constants. This procedure has been explained in Tack et al. (2014).

##### Glucose and oxygen uptake

Glucose and oxygen uptake are modeled according to the Monod kinetic model (Monod, 1942). A normally distributed stochastic element with a coefficient of variation of 0.10 has been superposed on this kinetic model, to incorporate biological variability and growth asynchrony (Schaechter et al., 1962; Koch, 1993).

##### Cell reproduction

The DNA replication and cell division processes are simulated according to an adapted version of the model of Donachie (1968), that has been developed in Tack et al. (2014, 2015). Daughter cells are placed along the orientation of their mother cell, upon which a uniformly-distributed random deviation angle of maximally π/8 radians is superposed.

##### Cell movement

Spatial overlap between neighboring cells is avoided by means of a cell shoving mechanism (Kreft et al., 1998). Detachment of cells from the biofilm's outer surface occurs when these cells are not properly aligned with their neighbors. Cell adhesion factors on the cell surface, such as Antigen 43 (Ag43) are mainly concentrated around the cell poles, implying that only the cell poles take part in intercellular adhesion interactions (Vejborg and Klemm, 2009). If a cell has less than four neighbors and is not attached to the substratum surface, it is assumed that this cell is situated at the biofilm exterior. To stay attached to the biofilm, the orientation vectors of this cell and one of its neighbors need to be aligned within a maximal detachment angle θ_*max*_ of π/6 with the line between the centers of these two cells.

#### Simulation results

##### Biofilm development

**Figure 3:**
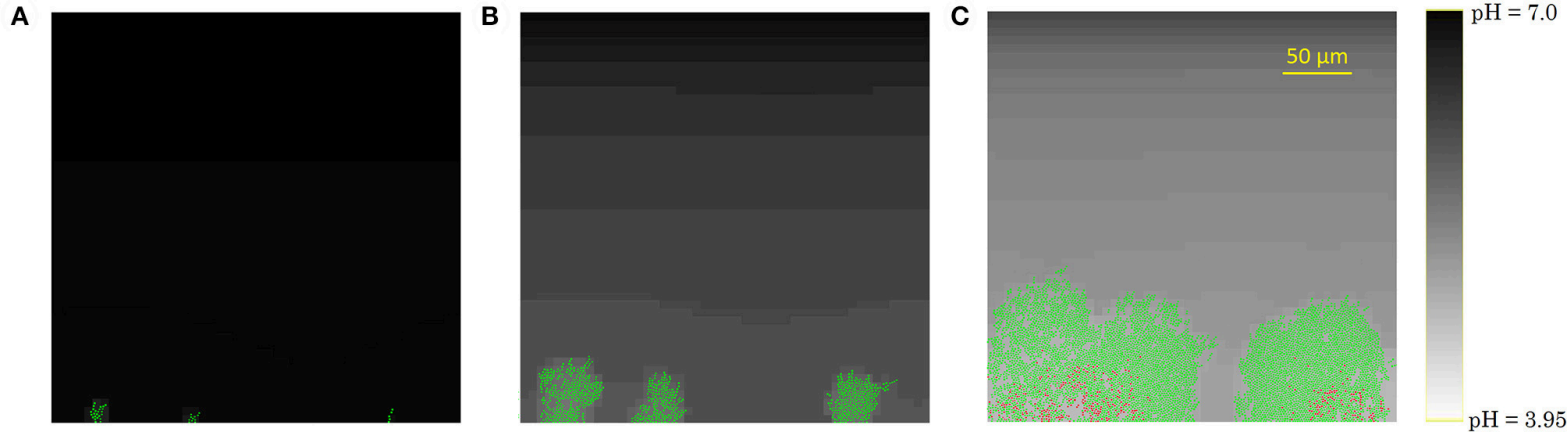
Evolution of the biofilm development: **(A)** cellular chain formation from the initial cells at the substratum surface at *t* = 10 h, **(B)** initial biofilm architecture at *t* = 25 h, **(C)** mature biofilm structure at *t* = 70 h. The viability of the cells is indicated by their color: green is used for actively growing cells, while red cells have stopped growing.

The development of the biofilm structure in the IbM simulation is presented in Figure 3. Initially, cellular chains form from the initial cells at the substratum surface, which has been experimentally observed (Vejborg and Klemm, 2009). After this initial stage, the biofilm environment gets oxygen-depleted and acidified at the substratum surface, mainly due to acetic acid production and to a lesser extent due to the formic acid secretion. Lactic acid production was not observed in the simulations as the oxygen concentration never dropped to a completely anaerobic level, which is a necessary condition for lactic acid secretion by the microbial cells. This acidification inhibits cellular growth and survival at the substratum, leading to a mature biofilm structure with mushroom-shaped pillars separated by water-filled channels. These channels are more acidified than the bulk medium, inhibiting cell growth. The mushroom-shaped architecture of mature biofilms has also been observed experimentally (Reisner et al., 2003).

Finally, due to the acidic cellular stress, cells stop growing at the substratum, indicated by the red cell color in Figure 3C. Ultimately, these growth-compromised cells die, resulting in biofilm detachment. Experimental studies of *E. coli* biofilm growth have demonstrated the failure of biofilm formation and the detachment of already existing biofilms under anaerobic conditions (Colón-Gonzáez et al., 2004). The absence of biofilm formation under anaerobic conditions has been explained by a reduced production of type 1 pili, inhibiting cell-substratum and cell-cell adhesion interactions. However, this explanation is not suitable for the detachment of already existing biofilms in which type 1 pili are abundantly available. This IbM simulation shows that acidification of the biofilm environment at the substratum surface can play a significant role in the biofilm detachment process as well.

##### Cell detachment

**Figure 4:**
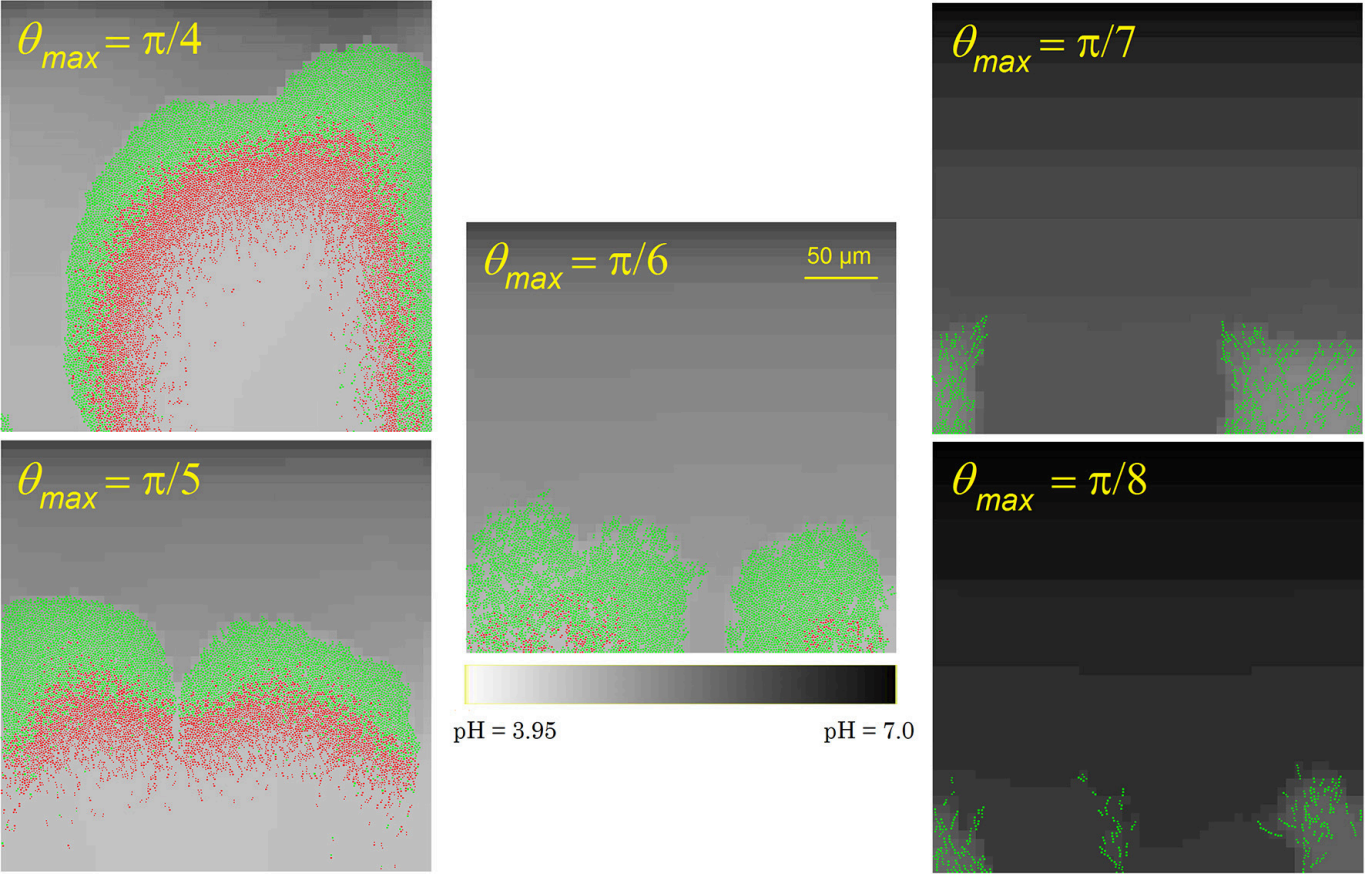
Biofilm structures at *t* = 70 h for different values of the maximal detachment angle θ_*max*_.

As the cell-cell adhesion factors on the cell surface are mainly concentrated around the cell poles, cells at the biofilm outer surface need to be well-aligned with their neighbors to avoid detachment from the biofilm due to flow shear forces. This results in the formation of cellular chains at the biofilm exterior surface (Vejborg and Klemm, 2009). To investigate the influence of the cell detachment process on the biofilm development and morphology, the maximal detachment angle θ_*max*_ is varied over a range of values. The results of this analysis are summarized in Figure 4. At higher values of θ_*max*_, i.e., less restrictive detachment conditions, a thicker biofilm structure with more densely packed cells emerges, causing more severe acidification and cell death at the substratum surface. Less cellular chains protrude from the exterior biofilm surface. For lower values of θ_*max*_, the opposite trend is observed as more cells detach from the biofilm and disperse into the bulk medium: the biofilm structure is thin and more open with many cellular chains. The biofilm structure at a maximal detachment angle of approximately π/6 corresponds best with experimental observations of *E. coli* biofilms (see e.g., Danese et al., 2000; Reisner et al., 2003; Vejborg and Klemm, 2009).

##### Initial cell affinity for the substratum surface

**Figure 5:**
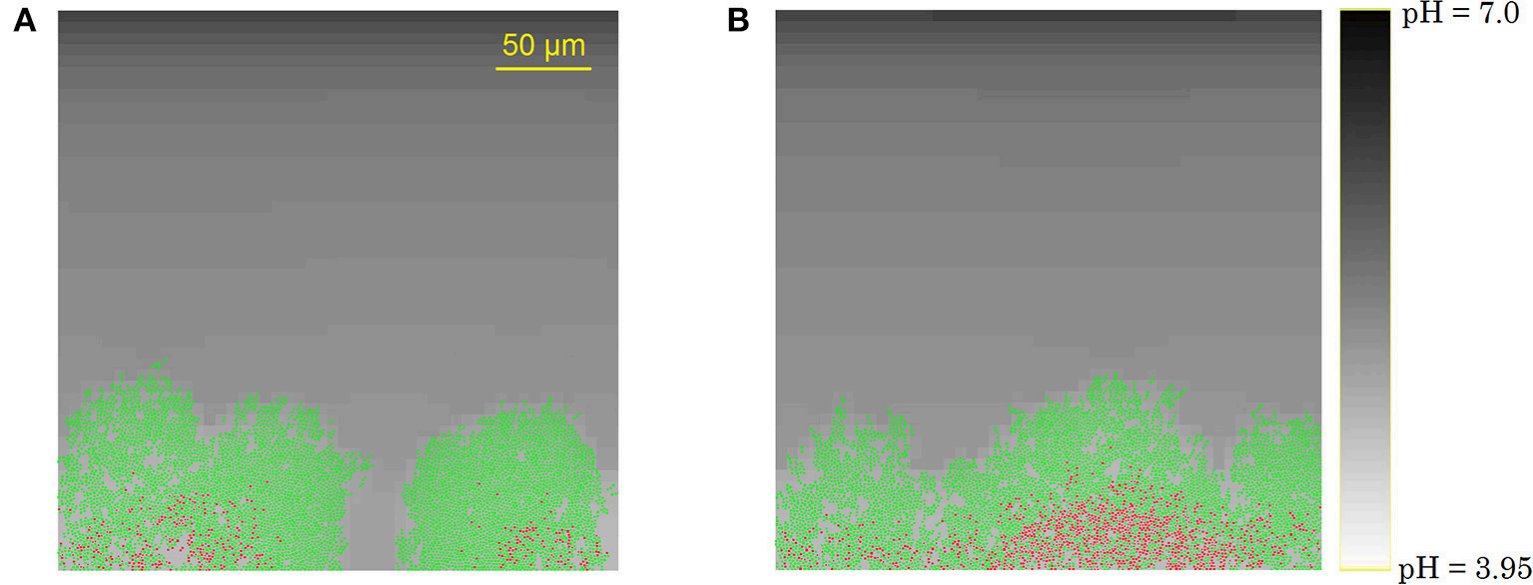
Biofilm structures at *t* = 70 h emerging from **(A)** 3 initial cells, or **(B)** 10 initial cells.

For the initial attachment of planktonic cells to the substratum, these cells need to overcome electrostatic repulsive forces from the substratum, that is often conditioned by the adsorption of various solutes to avoid biofilm growth. High affinities of dispersed cells in the bulk medium for the substratum lead to high densities of initial cells at the substratum surface. Increasing the number of initial cells at the substratum in the simulation leads to more continuous and flat biofilm structures, as illustrated in Figure 5. Both mushroom-shaped pillar structures for low initial cell numbers and more continuous structures at higher cell-surface affinities have been experimentally observed (see respectively, Reisner et al., 2003; Vejborg and Klemm, 2009). As a consequence, the substratum surface conditioning treatment plays a determining role in the formation of specific biofilm structures.

### Case study II: submerged colony growth

Most individual-based models for colony behavior are developed to describe phenomena that are experimentally observed for colonies on the surface of semi-solid food media. It is however much more difficult to experimentally observe dynamics of submerged colonies that are growing in mixed food products (Boons et al., 2001). For this reason, this case study focuses on submerged colony growth behavior and its connection to surface colony dynamics.

#### Model details

For the simulation of a submerged colony in semi-solid food products, the food system is modeled in principle as a three-dimensional environment to account for complex geometry as well, with the initial cell in the center. Note that the simulation of the growth of one submerged colony can be reduced to a two-dimensional problem in case of non-complex geometry. In such case, the two horizontal dimensions are equivalent, so only one horizontal dimension and the vertical dimension are needed if appropriate scaling of the spatial density of colonies from 3D to 2D is applied.

**Figure 6:**
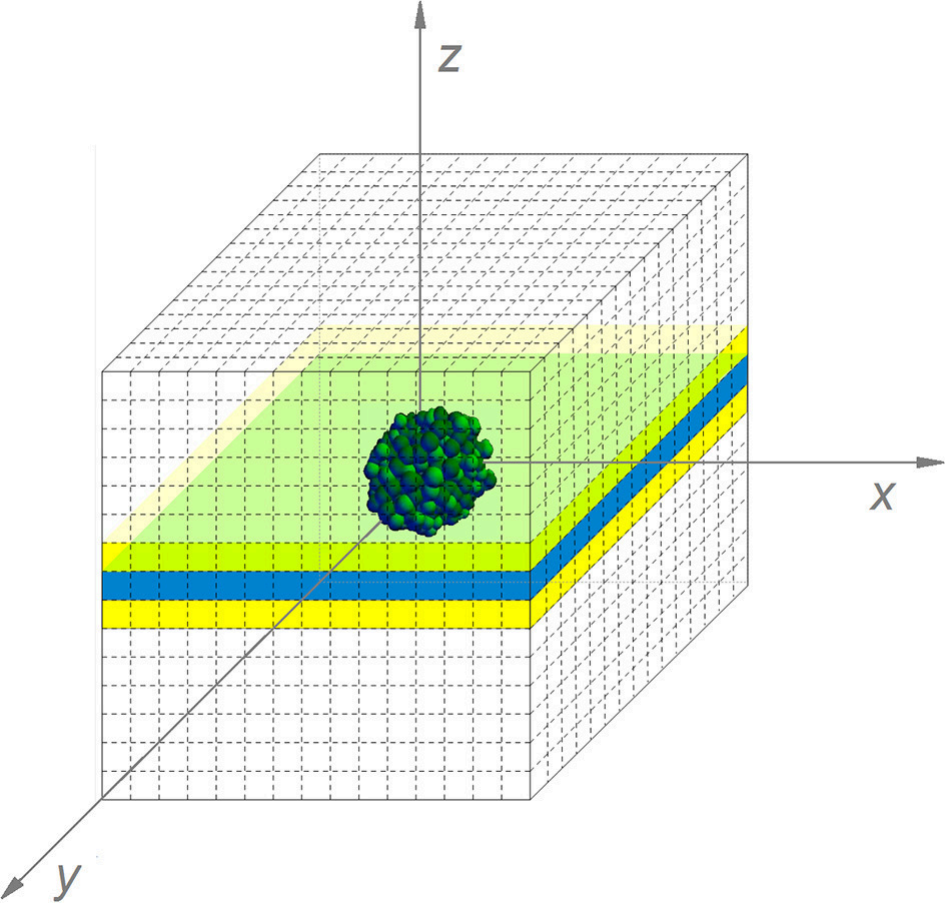
The simulated submerged colony environment (For reasons of clarity, the discretization pattern is represented in a more coarse way than in the real simulation).

The simulation of the diffusion processes in the environment cannot be reduced to a two-dimensional problem, as substances diffuse from or to the colony in the three spatial dimensions. However, updating the local concentrations in a complete 3D environment would be too computationally expensive. Therefore, only a central layer of the environment has been simulated, indicated in blue in Figure 6. Nevertheless, diffusion interactions in the perpendicular direction on this layer have been taken into account. For these interactions, the concentrations of environmental substances in the layers above and below the central layer, indicated in yellow in Figure 6, need to be determined. Under the assumption that the isoconcentration planes of chemical substances around the submerged colony can be locally approximated by concentric spheres around the environmental origin, the concentrations in the environmental units in the yellow layers are deducible from goniometric principles and interpolation between concentrations in the central layer. More specifically, the concentration of substance *i* in the yellow layers is expressed by means of the following expressions:

(15) $$\begin{array}{l} C_{i}(j,k,l-1)=C_{i}(j,k,l+1)=\\ (1-\text{sign}(\Delta_{y})\cdot \Delta_{y})\cdot \\ \;((1-\text{sign}(\Delta_{x})\cdot \Delta_{x})\cdot C_{i}(j,k,l)+\\ \text{ sign}(\Delta_{x})\cdot \Delta_{x}\cdot C_{i}(j+\text{sign}(\Delta_{x}),k,l))+\\ \text{sign}(\Delta_{y})\cdot \Delta_{y}\cdot \\ \;(\left(1-\text{sign}(\Delta_{x})\cdot \Delta_{x}\right)\cdot C_{i}(j,k+\text{sign}(\Delta_{y}),l)+\\ \text{ sign}(\Delta_{x})\cdot \Delta_{x}\cdot C_{i}(j+\text{sign}(\Delta_{x}),k+\text{sign}(\Delta_{y}),l)),\; \end{array}$$

where *C*_*i*_(*j, k, l*) is the concentration of substance *i* in the environmental unit with the coordinates (*j, k, l*), and

(16) $$\begin{array}{r} \Delta_{x}=\Delta \cdot \frac{j}{\sqrt{j^{2}+k^{2}}}, \end{array}$$

(17) $$\begin{array}{r} \Delta_{y}=\Delta \cdot \frac{k}{\sqrt{j^{2}+k^{2}}} \end{array}$$

(18) $$\begin{array}{r} \Delta =\sqrt{j^{2}+k^{2}+1}-\sqrt{j^{2}+k^{2}}, \end{array}$$

##### Initialization and boundary conditions

The simulation starts with one initial cell which is located in the origin of the environment. The environmental boundaries are characterized by constant chemical concentrations.

##### Diffusion

In the central environmental layer, diffusion processes are modeled according to the second law of Fick in three dimensions, discretized by means of the FTCS numerical scheme. For numerical stability reasons, a smaller time step Δ*t*_1_ = 0.00002 min is required in this explicit scheme for three-dimensional diffusion. For glucose and chemical compounds of the same molecular size as glucose, the diffusion coefficients in a 5% (w/v) agarose gel environment are approximately 75% of their normal diffusivities in water (Hooijmans et al., 1990; Andersson and Öste, 1994; Azevedo and Oliveira, 1995). The oxygen diffusivity is hardly effected by the agarose concentration (Guaccio et al., 2008).

##### Cell movement

As the cells cannot detach from the colony due to motility limitations, the spatial organization of the colony cells is only determined by cell shoving to avoid spatial overlap between neighboring cells.

#### Simulation results

**Figure 7:**
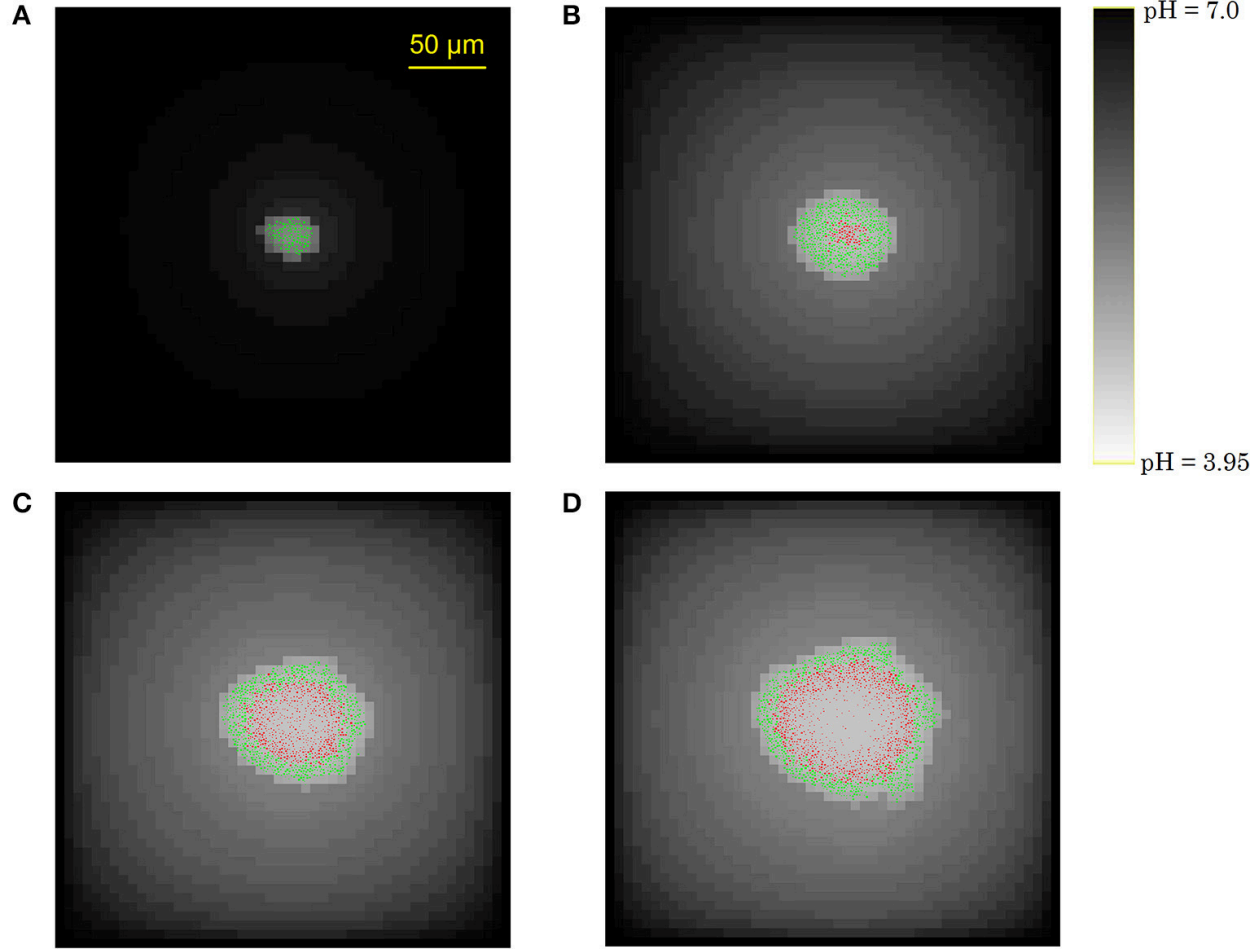
Evolution of the submerged colony development: **(A)** initial phase without severe growth-inhibiting conditions, **(B)** emergence of a central no-growth zone, **(C,D)** cell lysis in the colony center.

**Figure 8:**
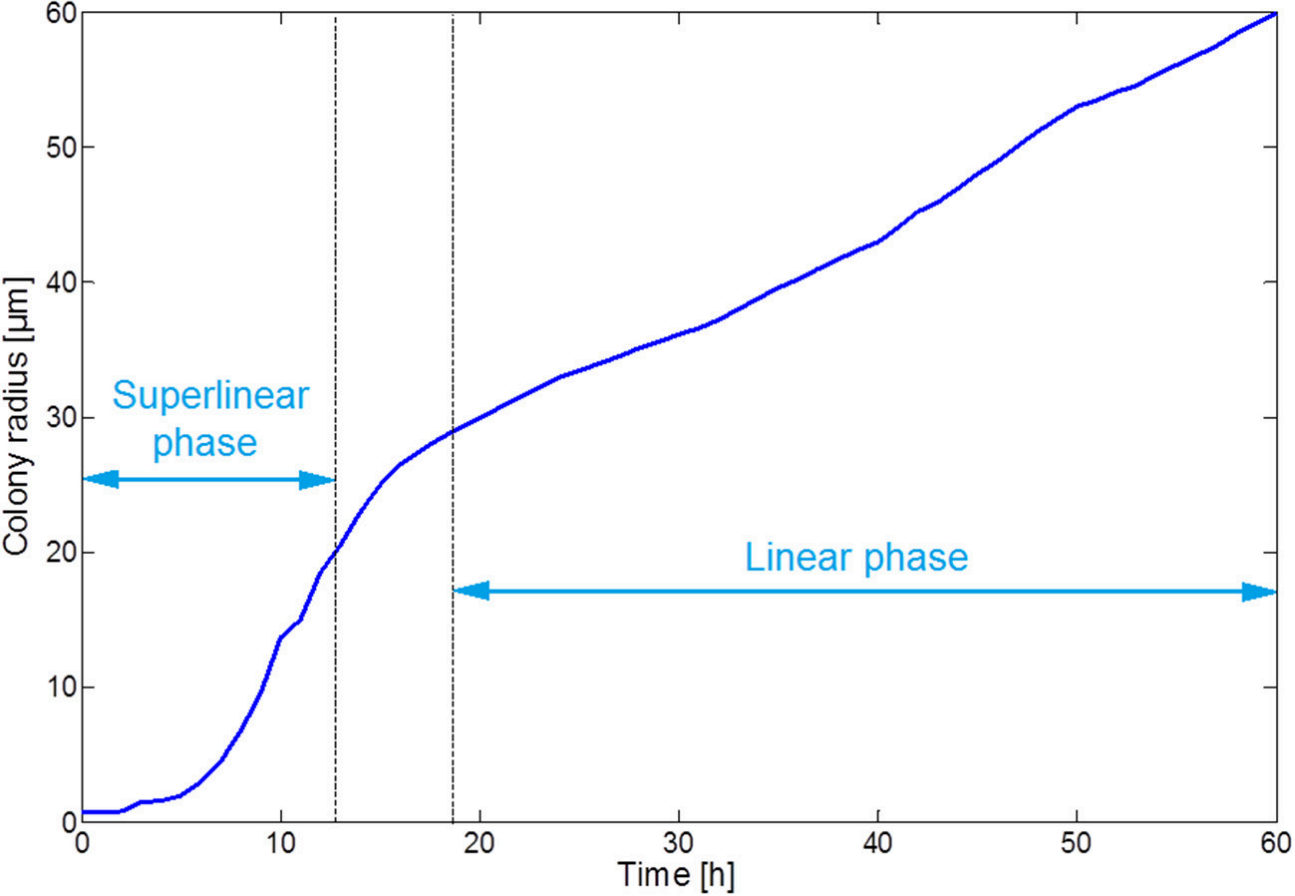
Evolution of the colony radius.

Figure 7 illustrates the growth of a submerged colony, starting from one initial cell in the origin of the environment. The colony remains more or less circular over its whole evolution, confirming the assumption that has been used to obtain the expressions in Equations (15–18). In the colony center, diffusion limitations lead to the accumulation of weak acid cell products, mainly acetic acid. The resulting pH drop causes cell lysis, indicated by the emergence of a no-growth zone and the disappearance of cells in the colony center. Due to the emergence of these no-growth conditions in the colony center, the initially superlinear growth of the colony radius slows down, resulting in a linear colony radius increase, as presented in Figure 8. This linear radius increase has been observed for quasi-two-dimensional surface colonies as well (Kamath and Bungay, 1988; Wimpenny et al., 1995; Mitchell and Wimpenny, 1997), indicating that easily-observable qualitative trends in two-dimensional surface colony dynamics can be representative for the behavior of three-dimensional submerged colonies that require much more advanced monitoring techniques.

## Discussion

An in-house developed simulator for individual based modeling of microbial dynamics has been extended with a metabolic model for *E. coli* expressing specific cellular growth rate and metabolic secretion rates as a function of the local extracellular pH and the concentration of undissociated cell products, covering all metabolic regimes from anaerobic respiration to anaerobic fermentation.

From the simulations, it is observed that *E. coli* biofilm dynamics are mainly determined by metabolic differentiation due to concentration gradients of weak acid cell products, cell detachment leading to persistent cell chains, and the affinity of planktonic cells for the substratum surface. Experimental studies also suggest a role for quorum sensing by the production or addition of autoinducer 2 (AI-2) signaling molecules, or through the production of *N*-acyl-L-homoserine lactones (AHL) by other species (DeLisa et al., 2001; Sperandio et al., 2001; González Barrios et al., 2006; Beloin et al., 2008). The effect of quorum sensing mechanisms on biofilm and microbial colony behavior has been investigated in other IbM simulations (see e.g., Tang et al., 2007; Nadell et al., 2008; Melke et al., 2010; Jang et al., 2012). In addition, cross-feeding interactions between the *E. coli* cells, such as the consumption of the produced acetate by cells at the substratum surface (Oh et al., 2002), has not been taken into account as this acetate uptake only occurs under low environmental concentrations of glucose. In the performed simulations, there is no local glucose depletion at the substratum surface. Furthermore, cells under stressing conditions may exhibit additional complex behavior and resilience by going into a dormant state (Agafonov et al., 2001). This transition to dormancy has not been taken into account in the simulations. The scope of this case study is in fact limited to the simulation of mature biofilm morphologies and the emergence of these morphologies is solely dependent on cellular events at the outer biofilm surface where the cells are not hibernated. Finally, it should be noted that the formation of cellular chains is a typical phenomenon in *E. coli* biofilms, which has not been observed for other species such as *Pseudomonas aeruginosa*. It is therefore not recommended to extrapolate the applied cell adhesion model to other species than *E. coli*.

The submerged colony simulations demonstrate that the initially homogeneous concentration of oxygen at saturation level decreases sharply over time in the colony center, while the oxygen concentration at the boundaries remains constant. However, it should be noted that these constant boundary conditions are a strong simplification of the conditions in real food systems, as the overall oxygen availability in food products strongly depends on the distance from the food surface (Noriega et al., 2008). The model may probably also be used for other food pathogens which are similar to *E. coli*, such as the gram-negative rod-shaped *Salmonella Typhimurium*. However, not enough microscopic data are available about this food pathogen to validate this assertion.

## Conclusions

Mature microbial communities of clustered cells, such as biofilms or colonies, are of paramount industrial and medical importance. Such microbial communities are characterized by metabolic differentiation among the constituting microorganisms due to diffusion limitations and chemical gradients in their environment. Due to the metabolic differences between the cells according to their position in the community, it is most appropriate to simulate these biosystems by means of an IbM with the microbial cell as basic modeling unit.

In this article, an in-house developed IbM platform for microbial dynamics, MICRODIMS, has been extended with a new metabolic model for the simulation of two-dimensional biofilm dynamics on abiotic food processing surfaces and three-dimensional submerged colony behavior in semi-solid food products. This metabolic model covers all metabolic regimes from aerobic respiration to anaerobic fermentation and expresses the specific cellular growth rate and metabolic secretion rates as a function of the local extracellular pH and the concentration of undissociated cell products. This model allows to study metabolic differentiation due to oxygen gradients in the development of *E. coli* cell communities, whereby low local oxygen concentrations lead to cellular secretion of weak acid products.

This metabolic model is expressed as a multiparametric programming problem, in which the influence of low extracellular pH values and the presence of undissociated acid cell products in the environment has been taken into account.

Two case studies have been elaborated in this article, using the MICRODIMS simulator: (i) biofilm growth on a substratum surface and (ii) submerged colony growth in a semi-solid mixed food product.

In the biofilm case study, accumulation of weak acid cell products and a concomitant pH drop occur at the substratum surface. This leads to cell lysis and biofilm detachment from the substratum. Apart from the metabolic cellular differentiation, biofilm dynamics are mainly determined by the cell detachment process at the biofilm outer surface, inducing the formation of protruding cell chains. The acidification of the biofilm environment and the emergence of typical mushroom-shaped morphologies of mature biofilms and the formation of cellular chains at the exterior surface of the biofilm are observed. In addition, high affinity of planktonic cells in the bulk medium for the substratum surface results in a high density of initial cells at the substratum and a more continuous and flat biofilm structure. The simulations show that these morphological phenomena are respectively dependent on the initial affinity of pioneer cells for the substratum surface and the cell detachment process at the outer surface of the biofilm.

The submerged colony case study demonstrates the development of a central no-growth zone with a sharp decline of the local pH, comparable to the pH drop at the substratum surface in the biofilm simulations. Cellular growth is limited to a thin band of cells at the colony periphery, resulting in a linear increase of the colony radius over time.

## Author contributions

Authors jointly designed the study and wrote the manuscript. IT implemented the algorithms and performed the computations. JV supervised the study. All authors have read and approved the final manuscript.

### Conflict of interest statement

The authors declare that the research was conducted in the absence of any commercial or financial relationships that could be construed as a potential conflict of interest.
